# Influence of light and shoot development stage on leaf photosynthesis and carbohydrate status during the adventitious root formation in cuttings of *Corylus avellana* L.

**DOI:** 10.3389/fpls.2015.00973

**Published:** 2015-11-06

**Authors:** Sergio Tombesi, Alberto Palliotti, Stefano Poni, Daniela Farinelli

**Affiliations:** ^1^Dipartimento di Scienze Agrarie, Alimentari e Ambientali, Università di PerugiaPerugia, Italy; ^2^Dipartimento di Scienze delle Produzioni Vegetali Sostenibili, Università Cattolica del Sacro CuorePiacenza, Italy

**Keywords:** hazelnut, soft wood cuttings, rooting, carbohydrates, light

## Abstract

Adventitious root formation in plant cuttings is influenced by many endogenous and environmental factors. Leaf photosynthesis during rooting of leafy cuttings in hard to root species can contribute to supply carbohydrates to the intensive metabolic processes related to adventious root formation. Light intensity during rooting is artificially kept low to decrease potential cutting desiccation, but can be limiting for photosynthetic activity. Furthermore, leafy cuttings collected from different part of the shoot can have a different ability to fuel adventitious root formation in cutting stem. The aim of this work was to determine the role of leaf photosynthesis on adventitious root formation in hazelnut (*Corylus avellana* L) (a hard-to-root specie) leafy cuttings and to investigate the possible influence of the shoot developmental stage on cutting rooting and survival in the post-rooting phase. Cutting rooting was closely related to carbohydrate content in cutting stems during the rooting process. Cutting carbohydrate status was positively influenced by leaf photosynthesis during rooting. Non-saturating light exposure of leafy cuttings can contribute to improve photosynthetic activity of leafy cuttings. Collection of cuttings from different part of the mother shoots influenced rooting percentage and this appear related to the different capability to concentrate soluble sugars in the cutting stem during rooting. Adventitious root formation depend on the carbohydrate accumulation at the base of the cutting. Mother shoot developmental stage and leaf photosynthesis appear pivotal factors for adventitious roots formation.

## Introduction

Adventitious root formation in plant cuttings is a complex physiological process involving plant growth substances as well as cutting water relations and nutritional status (Hartmann and Kester, 1983). The understanding of the physiological bases underlying adventitious root formation is essential for the selection of plant material to be used for cutting, the cutting preparation prior to rooting and choice of best environmental conditions during cutting rooting. These factors have a primary influence on rooting percentage that is crucial for economic viability of hard-to-root species propagation.

Adventitious root initiation and growth is an intensive metabolic process that, promoted by auxins and/or other promoting growth regulators, lead to the increase of enzyme activity and to the synthesis of RNA and proteins (Hartmann and Kester, 1983; DeKlerk et al., 1999; Legué et al., 2014). Starch content in cutting stem decreases during root formation, providing energy, and carbon skeletons to the rooting zone and fueling root development and growth (Hartmann and Kester, 1983; Haissig, 1989; Druege et al., 2004). Cutting leaves assimilate CO_2_ over the whole rooting phase (Smalley et al., 1991; Yue and Margolis, 1993; Klopotek et al., 2012) even though in pea and *Acer rubrum* cuttings photosynthesis is limited (Davis and Potter, 1981; Smalley et al., 1991). Thus, leaf photosynthesis in leafy cuttings can play a crucial role at providing carbohydrates to root formation and growth in particular when the rooting process lasts for several weeks.

High light intensity is considered detrimental for cutting rooting because it increases leaf temperature and transpiration and facilitates leaf and cutting dehydration. In fact, high light regimes decrease cutting rooting in many woody and herbaceous species (Stoutemyer and Close, 1946; Waxman, 1965; Hansen, 1975; Loach, 1979; Loach and Gay, 1979; Grange and Loach, 1985; Aminah et al., 1997; Zaczek et al., 1997; Zobolo, 2010). On the other hand, in some other species—especially herbaceous—daily light irradiation is positively correlated with cutting rooting and development (Lopez and Runkle, 2008; Park et al., 2011; Currey et al., 2012). If carbohydrate pool of cuttings can influence cutting rooting, then sustained leaf photosynthesis could contribute to replenish or to increase cutting carbohydrate content. In greenhouses, light intensity, the main driver of leaf photosynthesis, is generally kept low (i.e., well below the light saturation point for photosynthesis) to limit leaf transpiration and heating.

Hazelnut (*Corylus avellana* L.) is considered a species difficult to be propagate by cutting (Hartmann and Kester, 1983). Currently, hazelnut is propagated mainly by layering, but the large number of mother plant required for layering (i.e., transmission of bacteria and viruses) make it difficult to assure the sanity of propagation material. On the other hand, hazelnut grafting is still not a common practice due to the erratic graft taking percentage and the higher cost of this propagation system as compared to layering. However, hazelnut propagation by cutting is viable, and recent studies have reported rooting percentages variable from 20 to 70% depending on the cutting treatment and the time cuttings are collected (Cristofori et al., 2010; Contessa et al., 2011). In particular, timing of cutting collection is a crucial factor for cutting rooting in hazelnut; the best period of the year is June in the north hemisphere (Cristofori et al., 2010) and January in the south hemisphere (Santelices and Palfner, 2010). Over that period of the year vegetative shoots are still growing and shoot lignification is starting to occur from the base to the apex. Earlier reports on other species have pointed out that cuttings taken from different part of the shoots (i.e., apical or basal) can have different rooting capability; for instance, blueberry (*Vaccinum corimbosum* L.) and *Peltophorum pterocarpum* cuttings taken from the basal portion of shoots rooted much better than those taken from the terminal part (O'Rourke, 1944; Saifuddin et al., 2013), whereas the opposite occurred in cherries (*Prunus avium* L.) (Hartmann and Brooks, 1958). Our first hypothesis was that cutting leaf photosynthetic activity under greenhouse condition is similar to that measured in the field and that a moderate increase of light availability in greenhouse could increase leaf photosynthesis in cuttings leading to an increase of carbohydrate content in leafy cuttings stimulating root formation and growth. We hypothesized that the use of mist sprays can control the water status of leaf cuttings by counterbalancing the increased temperature and the increased leaf transpiration. Furthermore, considering that in hazelnut the optimal cutting collection time is quite limited and that it coincides with active vegetative shoot growth, our second hypothesis was that the development stage of cuttings can influence their rooting capability and their subsequent survival in the post-rooting phase.

The aim of the present work was to determine the role of leaf photosynthesis on adventitious root formation in hazelnut leafy cuttings and to investigate the possible influence of the shoot developmental stage on cutting rooting and survival in the post-rooting phase.

## Material and methods

### Mother plants

Cuttings of *Corylus avellana* L cv. Tonda di Giffoni were collected in 2014 at the experimental orchard of the Department of Agricultural, Food, and Environmental Sciences of the University of Perugia located nearby Deruta, Italy (42°58′21.8″N, 12°24′08.7″E). Mother plants were 30 year old and were trained to free bush. A topping cut at 1 m of height was carried out during January 2014 by a disk pruning machine to stimulate emissions of vegetative suckers to be used as mother shoots. Cuttings were harvested from the vegetative shoots originated from the part immediately below the pruning cut.

### Gas exchanges and water potential in mother plants

Prior to cutting collection, photosynthetic light response curves were measured between 10 and 12 a.m. on 10 leaves per each shoot zone (i.e., basal, between node 2 and 7, apical, between node 8 and 13) using a portable open system LCA3 infrared gas analyser (ADC Bioscientific ltd., Hoddesdon, UK) equipped with a Parkinson leaf chamber (11.2 cm^2^ area). Leaves were adapted to dark for 20 min, then gas exchange measurements started and leaves were progressively exposed to five levels of PAR by covering the leaf by a shield composed by a variable number of shading net sheets Maximum quantum yield was obtained by the slope of the regression equation of An vs. PAR at PAR values below saturation point (PAR < 500 μmol m^−2^ s^−1^) After the A_n_ vs. PAR measurement, Ψ_stem_ was measured on each mother plant (*n* = 5) on one mature leaf that had been wrapped in plastic film and aluminum foil 2 h prior to the measurements (McCutchan and Shackel, 1992) using a pressure chamber (Soilmoisture Corp, Santa Barbara, CA, USA). Ψ_leaf_ was measured as previously described on other five leaves per each shoot portion after the A_n_ vs. PAR measurements.

### Cutting collection and rooting conditions

On June 23rd shoots were cut above the second basal node, immediately placed in a container with the basal part dipped in tap water and then taken to the greenhouse facility close the orchard. At this point shoots had 12–13 nodes; shoot apices (terminal 3 nodes) were discarded, then mother shoots were cut in two segments (basal and apical part of the shoot) of about five nodes each. Single node leafy cuttings (7–10 cm long) were obtained sectioning basal and apical mother shoots segments.

Cutting base was immediately dipped in 500 mg L^−1^ hydro-alcoholic solution (80/20 v.v.) of Indol-Butyric Acid (IBA) for 60 s (Contessa et al., 2011). IBA-treated cuttings were immediately placed in a rooting bench at a density of ~300 cuttings/m^2^. About 1800 cuttings (half from basal and half from apical part of mother shots) were used in the experiment. The rooting bench (1.20 m wide and 6 m long) was equipped with a bottom heat apparatus set at ~25°C and a mist system. Mist nozzles were placed every 1 m in the center of the rooting bench. Mist duration and frequency during the daylight time was set at 15 s and 15 min, respectively. Perlite was used as rooting medium.

Three different light regimes were applied during the rooting period (i.e., June 23–August 8) described as follows: Control, greenhouse light intensity (~100 μmol m^−2^ s^−1^); Light+, greenhouse light intensity plus artificial lighting (Son-T Agro 400 W, Philips, Amsterdam, NL) from 7 a.m. to 7 p.m. (~200–300 μmol m^−2^ s^−1^); Shaded, the bench was covered with a shading net (~30% absorbed light) placed at 30 cm above the cutting leaves and allowing a light intensity ~30–70 μmol m^−2^ s^−1^. About 300 basal and 300 apical cuttings were subjected to each light regime.

### Gas exchange and water potential in cuttings

Air temperature and relative humidity (RH) was measured in each light treatment every 60 s by a temperature and humidity data logger (PCE-HT71, PCE, Italy) during all the rooting period. Air vapor pressure deficit (VPD) was calculated after Monteith and Unsworth (1990).

Stomatal conductance (g_s_) and net assimilation (A_n_) measurements were carried out between 10 and 12 a.m. every three days during the first 10 days and, hereafter, approximately every week on five leaves (i.e., five cuttings) per treatment using a portable open system LCA3 infrared gas analyser (ADC Bioscientific ltd., Hoddesdon, UK) equipped with a Parkinson leaf chamber (11.2 cm^2^). Prior measurements, cutting leaves superficies were gently soaked up by a dry paper to eventually remove free water on leaf surface. Measurements were performed under current light intensity at the timing of measurement maintaining each leaf in its current position. Cutting leaf water potential (Ψ) was measured over the same days and immediately after gas exchange measurements using a pressure chamber (Soilmoisture Corp, Santa Barbara, CA, USA).

### Carbohydrate determination

Cuttings used for gas exchange (*n* = 5) and Ψ measurements were immediately placed in liquid nitrogen and then stored in a freezer at −80°C. Then, the material was weighted and lyophilized (LIO5P, 5 Pascal, Trezzano, Italy). Lyophilized material was weighted (dry weight) and grinded (MF10, IKAlabortechnik, Staufen, Germany). Leaf material and basal portion (basal 5 cm) of cutting stem 0.01 g of powder were placed in 15 ml tubes and added with a solution of ethanol 80% and placed in a warm bath with temperature set at 80°C for 1 h. After 10 min of centrifugation at 10,000 rpm, 10 μl of supernatant was sampled and used for the determination of alcohol soluble sugars by the Anthrone method (Morris, 1948; Loewus, 1952). For starch determination, pellet material was then washed with sodium acetate buffer and then added with 0.5 ml of sodium acetate buffer. Tubes were placed in warm bath with temperature set at 80°C for 1 h. One milliliter of solution of amyloglocosidase and α-amylase in 0.05 M sodium acetate buffer was added as described by Chow and Landhäusser (2004) and bath temperature was set at 50°C. Sugar content was then measured on the supernatant by the anthrone method as previously described

### Cutting rooting assessment and post-rooting cares

Rooting was assessed two times during the experiment: July 15 [Day of the year (DOY) 194] and August 8 (DOY 214). At DOY 194 cuttings were separated in dead (blackened and rot cuttings), still (without any visual symptom of rooting), callused (with callus on the basal part of the stem), and rooted (with at least one visible root) in order to assess if the treatments had effect on the speed of adventitious root formation. At the end of the experiment cuttings were reclassified in dead, poorly rooted (sum of total root length shorter than 10 cm), well-rooted (sum of total root length longer or equal to 10 cm).

On August 8, after rooting assessment, cuttings were transferred in 4 L pots filled with a mixture of peat:pozzolana (50:50 v.v.) and taken back to the greenhouse under a light regime similar to that of the control treatment. Cuttings were tagged and divided in different light treatments, cutting types (apical, basal) and rooting quality (well and poorly rooted). Pots were placed on another bench equipped with a mist system. Mist duration and frequency during the daylight time was set to 15 s and 60 min, respectively to prevent leaf desiccation (Fordham et al., 2001) and plant mortality was assessed on March 31, 2015 after sprouting in order to evaluate the possible economic impact of the tested techniques.

### Statistical analysis

Statistical analysis was performed using linear and non-linear regression analysis to assess the correlation between PAR and A_n_ and the correlation between carbohydrates and rooting percentage using Sigmaplot 8.0 (Systat Software Inc., San Jose, CA, USA) and R^2^ significance was assessed by ANOVA. Treatments were analyzed by One-way ANOVA with significance level set at 0.05; means were separated by Tukey's w-procedure at *P* = 0.05. Relative frequencies were analyzed by χ^2^-test. *P*-value was set at 0.05.

## Results

**Figure 1:**
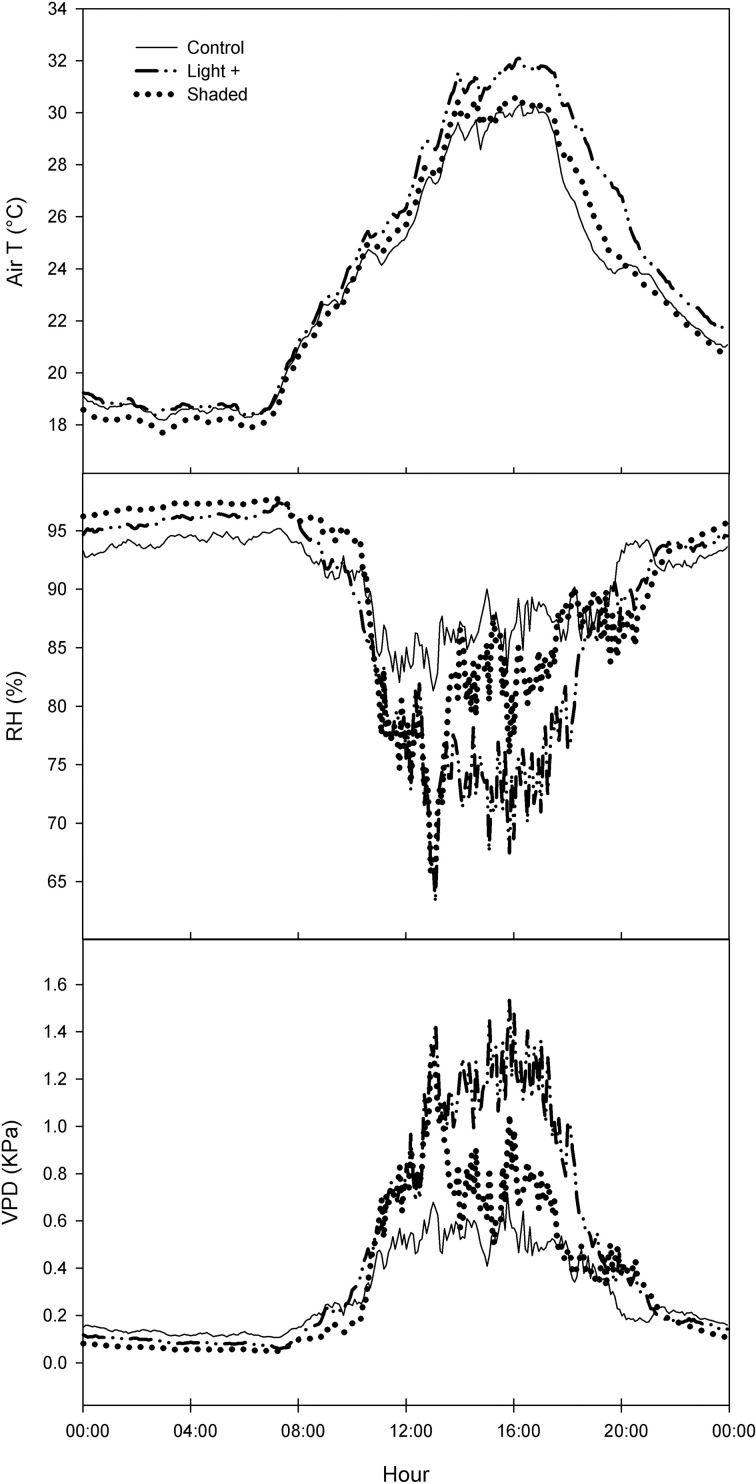
**Daily trend of air temperature (T), relative humidity (RH), and air vapor pressure deficit (VPD) in Control, Light+, and Shaded treatments**. Curves are the mean of three consecutive days (DOY 174–176).

Light+ treatment caused an increase of air temperature of about 2°C in the first afternoon in comparison with other two treatments (Figure 1). RH was lower in Light+ treatment of about 10% points than in other two treatments. Differences in air temperature and RH resulted in V_air_ increase between 0.4 and 0.5 KPa in the Light+ treatment in comparison with the other two treatments during the afternoon.

**Figure 2:**
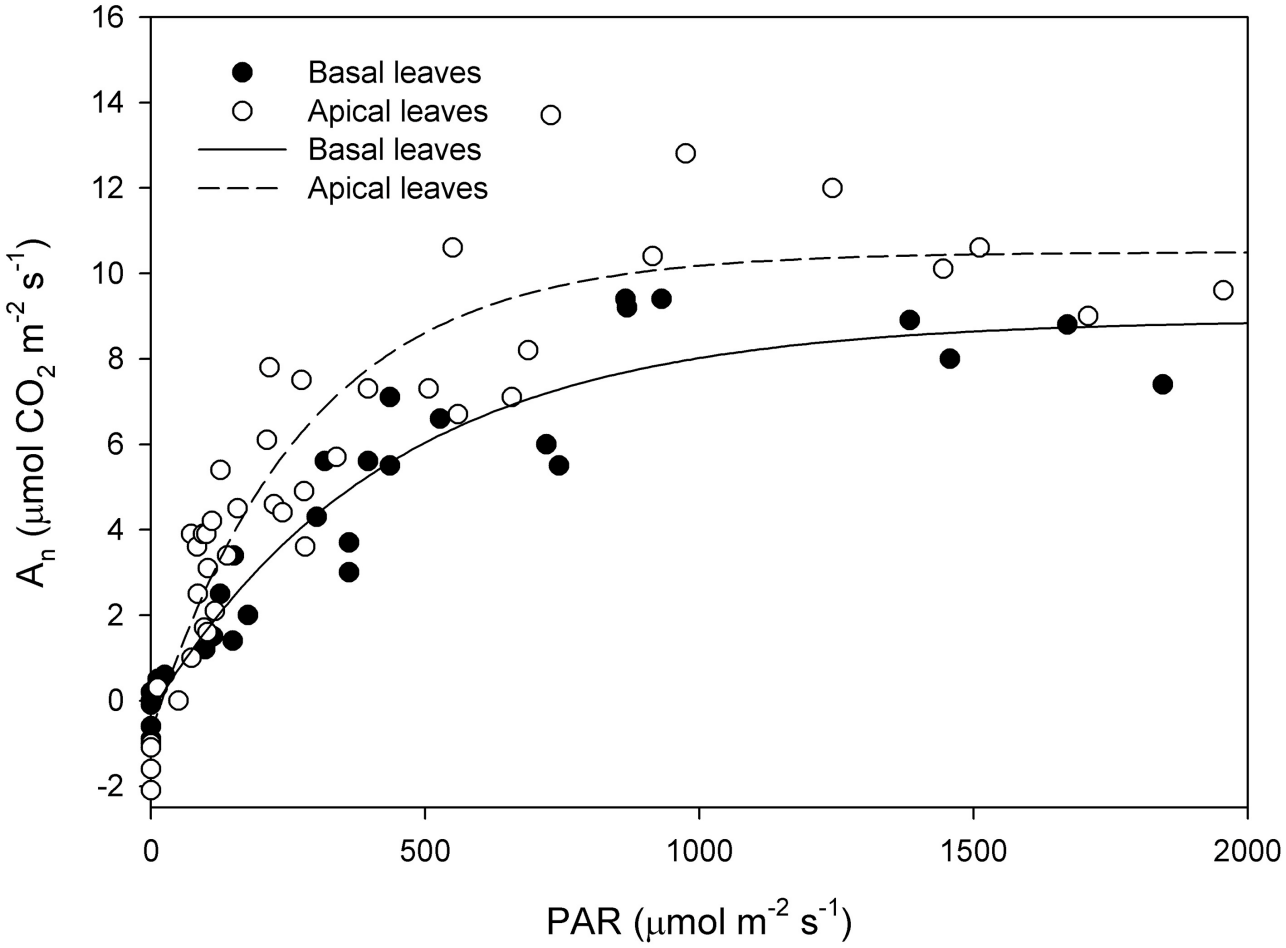
**Net CO_2_ assimilation (A_*n*_) vs. Photosynthetic Active Radiation (PAR) in basal and apical shoot leaves measured in the field on the collecting date**. [basal leaves *y* = −0.28+9.2(1-e^−0.002x^) *R*^2^ = 0.92 *P* < 0.001; apical leaves *y* = −0.78+11.27(1-e^−0.003x^) *R*^2^ = 0.87 *P* < 0.001].

**Figure 3:**
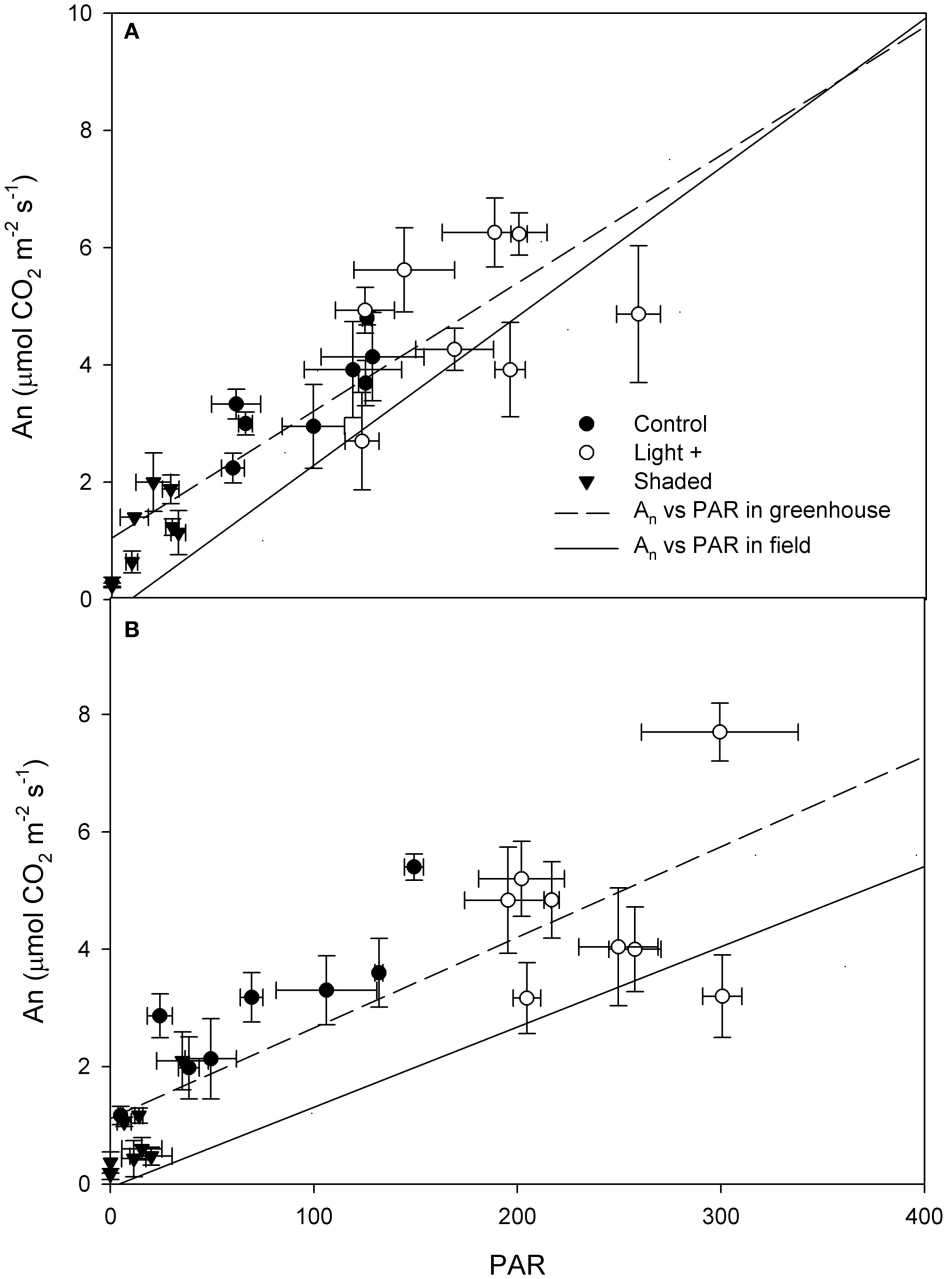
**Net assimilation (A_*n*_) vs. PAR measured on cutting leaves collected from the shoot apical part (A) and basal part (B) during the rooting period in the greenhouse**. Dashed line represent the linear regression (apical *y* = 0.02x + 1.05, basal *y* = 0.015x + 1.11) of measurements carried out in greenhouse over the rooting period (DOY 172–214). Solid line represent the regression (apical *y* = 0.025x − 0.22, basal *y* = 0.013x − 0.055) of measurements carried out on mother plants in field prior cutting collection. Each point is the mean of five leaves ±SE.

Photosynthetic light-response curve in the field was significantly different between basal and apical shoot leaves (Figure 2) as the latter had higher A_n_ at saturating light (basal 7.92 ± 0.65 μmol CO_2_ m^−2^ s^−1^, apical 9.85 ± 0.95 μmol CO_2_ m^−2^ s^−1^). Than basal shoot leaves (mean A_n_ at saturating light (PAR > 500 μmol photons m^−2^ s^−1^) was compared by *t*-test *P* < 0.01). Quantum yield (Φ) was significantly lower in basal shoot leaves than in apical shoot leaves (apical Φ = 0.025 μmol CO_2_ μmol photons^−1^; basal Φ = 0.013 μmol CO_2_ μmol photons^−1^) (Figure 3). A_n_ measured in cuttings collected from shoot apical and basal parts (Figures 3A,B, respectively) was correlated with PAR (apical *R*^2^ = 0.77 *P* < 0.001; basal *R*^2^ = 0.69 *P* < 0.001). Regressions slope of A_n_ vs. PAR measured in greenhouse during rooting was similar to that measured in the field prior cutting collection (test for homogeneity of regression apical *P* = 0.42, basal *P* = 0.44).

**Figure 4:**
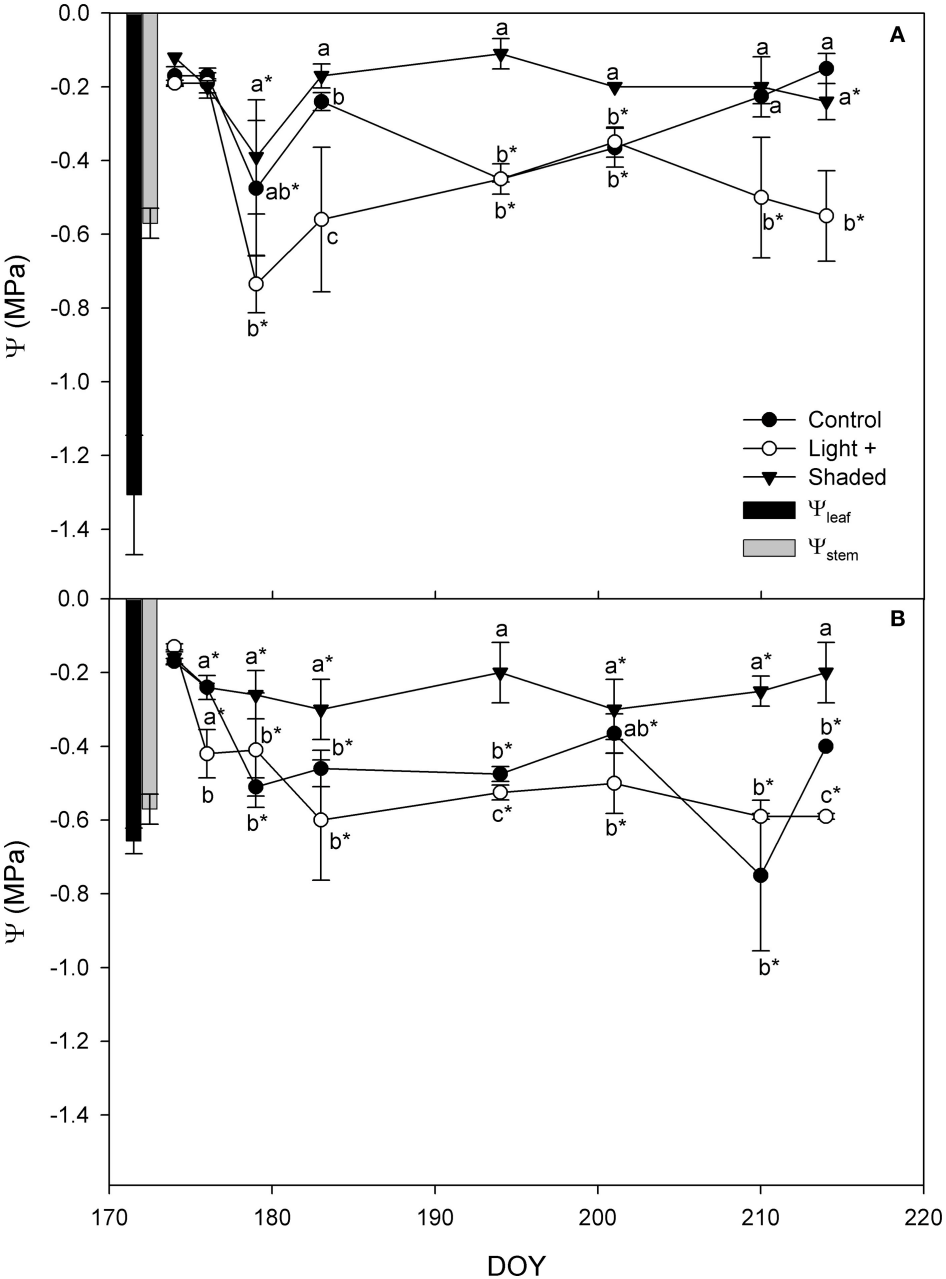
**Cutting water potential in Control, Light+, and Shaded cuttings collected from the shoot apical part (A) and basal part (B) during the rooting period in the greenhouse**. Columns depict Ψ_stem_ and Ψ_leaf_ measured in the field on mother plants in the cutting collection day at 12 a.m. Each point is the mean of five leaves ± SE. Points of the same sampling date with different letters are different per *P* = 0.05 (Tukey test). Point marked with asterisk are different from the value measured on the cutting collection day per *P* = 0.05 (Tukey test).

Shaded cuttings had higher Ψ while Light+ had the lowest Ψ values throughout the experiment in both apical and basal portions (Figure 4). Control cuttings had Ψ values in between the other two treatments. However, apical cutting Ψ values measured during the rooting were consistently higher than leaf water potential measured in the field prior cutting collection. In basal cuttings the difference between cutting Ψ and Ψ_leaf_ was less marked, although cutting Ψ was never below the Ψ_leaf_ during rooting.

**Figure 5:**
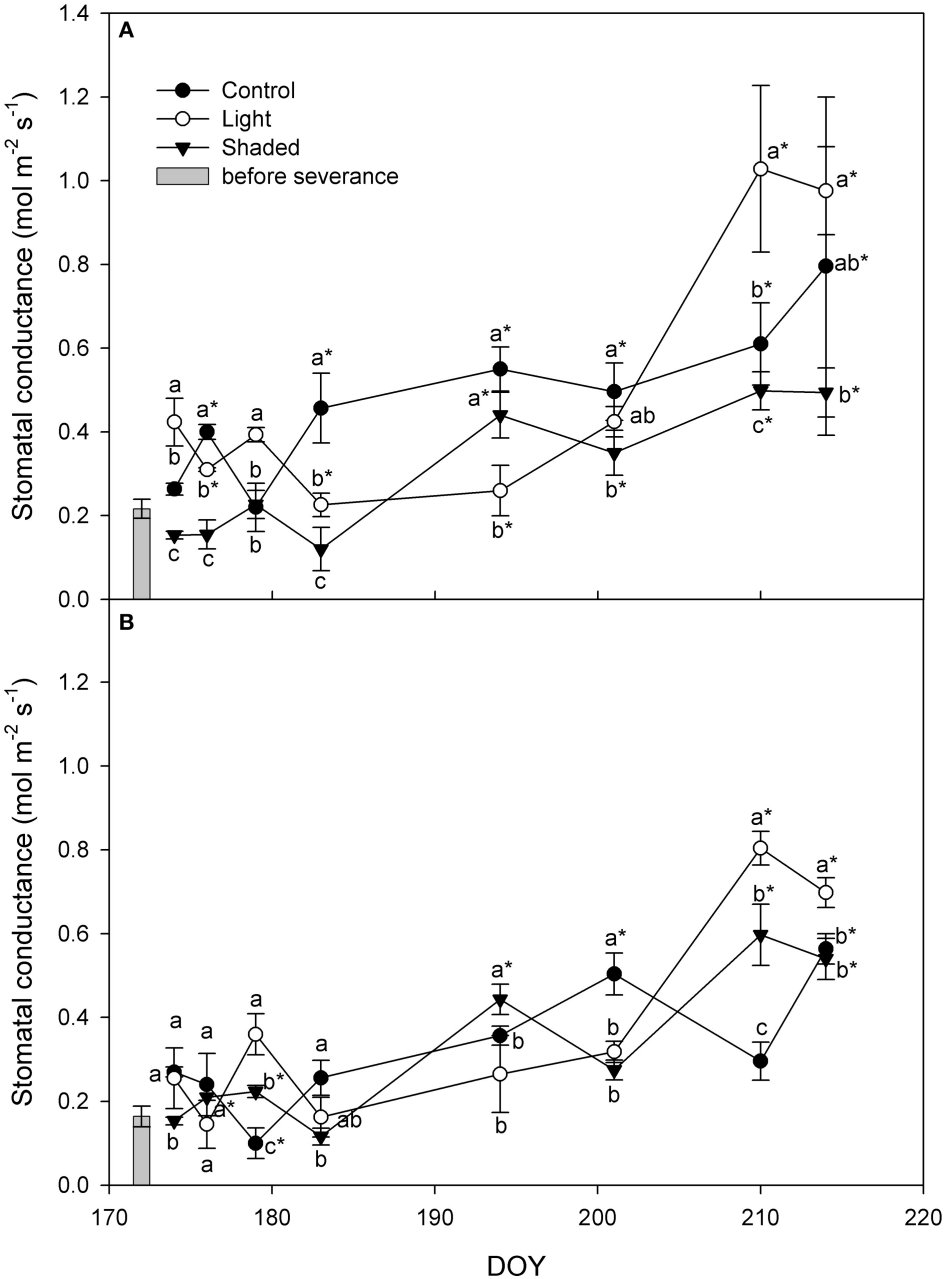
**Leaf stomatal conductance (g_s_) in Control, Light+, and Shaded cuttings collected from the shoot apical part (A) and basal part (B) during the rooting period in the greenhouse**. Columns depict g_s_ measured in the field on mother plants in the cutting collection day at 12 a.m. Each point is the mean of five leaves ± SE. Points of the same sampling date with different letters are different per *P* = 0.05 (Tukey test). Point marked with asterisk are different from the value measured on the cutting collection day per *P* = 0.05 (Tukey test).

Stomatal conductance (g_s_) increased over the whole rooting period in all treatments in basal as well as in apical cuttings (Figure 5). Moreover, g_s_ started to increase right after cuttings were placed in greenhouse. At the end of the experiment Light+ rooted cuttings had higher g_s_ than Shaded cuttings in basal and apical cuttings. Control cuttings had g_s_ value in between those of Light+ and Shaded cuttings.

**Figure 6:**
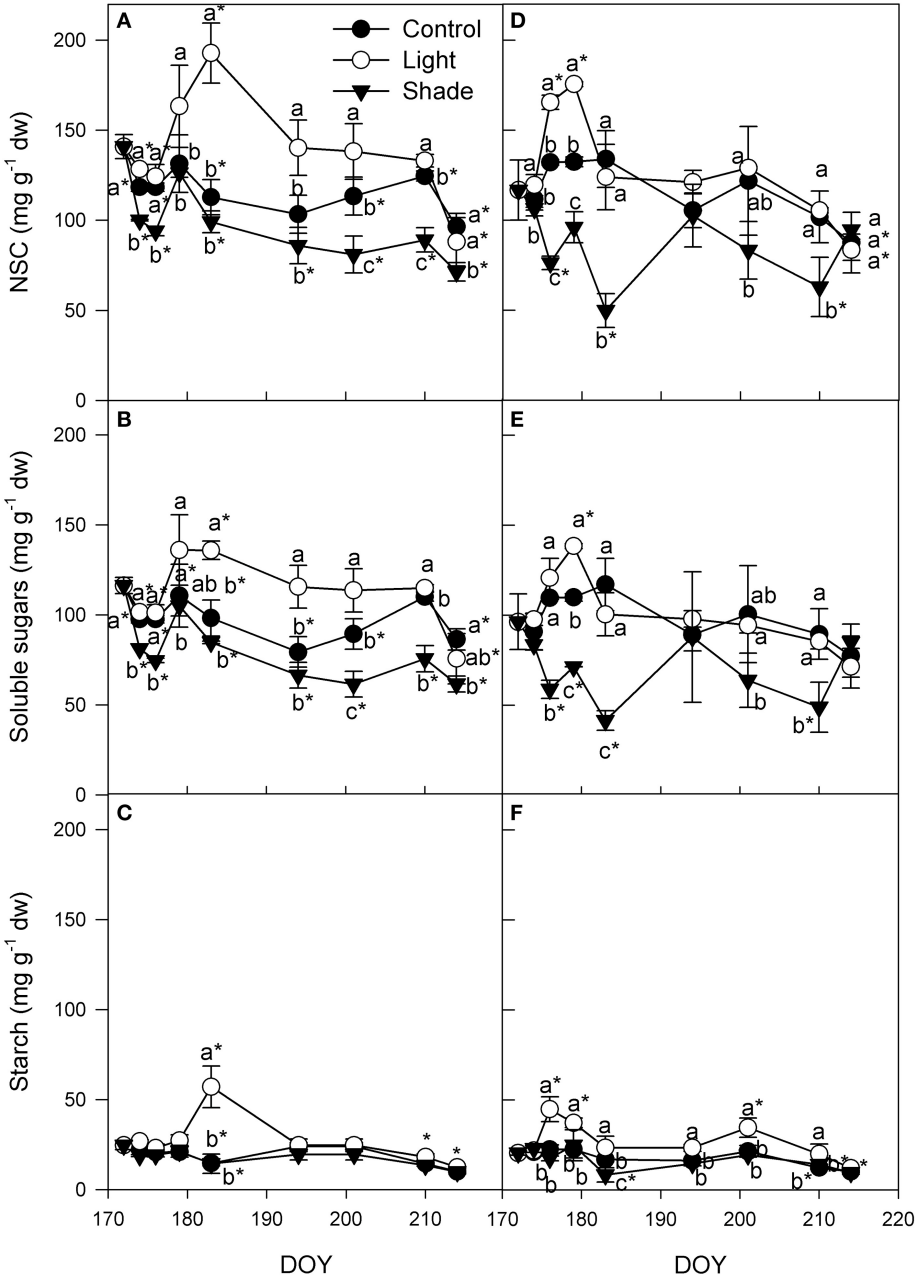
**Non-structural carbohydrates (NSC), soluble sugars and starch in leaves of cuttings collected from shoot apical part (A,B,C) and basal part (D,E,F)**. Each point is the mean of five leaves ± SE. Points of the same sampling date with different letters are different per *P* = 0.05 (Tukey test). Points without letters were similar per *P* = 0.05. Point marked with asterisk are different from the value measured on the cutting collection day per *P* = 0.05 (Tukey test).

Leaf non-structural carbohydrates (NSC) content in apical cutting leaves slightly decreased in the first week after cutting collection in Light+ and control apical cuttings (Figure 6). After the first week, NSC in leaves of cuttings collected from the apical part of the shoot of Control and Shaded treatment was almost steady up to DOY 210. In the last sampling at DOY 214, NSC decreased in all three treatments. Opposite to the pattern of the other two treatments, in Light+ cuttings collected from the apical part of the shoot, leaf NSC content increased up to DOY 184 and thereafter decreased up to the end of rooting period. NSC pattern in all treatment reflected that of soluble sugars because during the whole experiment leaf starch content remained almost constant except for DOY 183 when Light+ leaves had a significant higher starch content than other two treatments and original content at cutting collection date (Figures 6B,C). In cuttings collected from the basal part of the shoot, leaf NSC content increased during the first 10 days in the Light+ treatment (Figure 6D). In Control treatments, leaf NSC was stable across the same period and in Shaded treatment leaf NSC content decreased. At DOY 183 leaf NSC content rapidly decreased in Light+ treatment and remained almost steady up to the end of the experiment even if during this time span leaf NSC showed a slightly decreasing trend. After DOY 183, Control cuttings collected from the basal part of the shoot had a NSC content in leaves similar to that of Light+ cuttings. On the other hand, Shaded cuttings collected from the basal part of the shoot, even though with some fluctuations, maintained an almost steady level of NSC from DOY 179 up to the end of the experiment. As for the cuttings collected from the apical part of the shoot, also in cuttings collected from the basal part of the shoot, NSC pattern in leaves was mainly the results of soluble sugar variation (Figure 6E). Leaf starch was almost steady all over the experiment even though Light+ basal leaves had higher starch content than other two treatments from DOY 174 up to DOY 210 (Figure 6F). At DOY 174, 179, and 201 leaf starch content in light+ treatment was higher than that measured at cutting collection.

**Figure 7:**
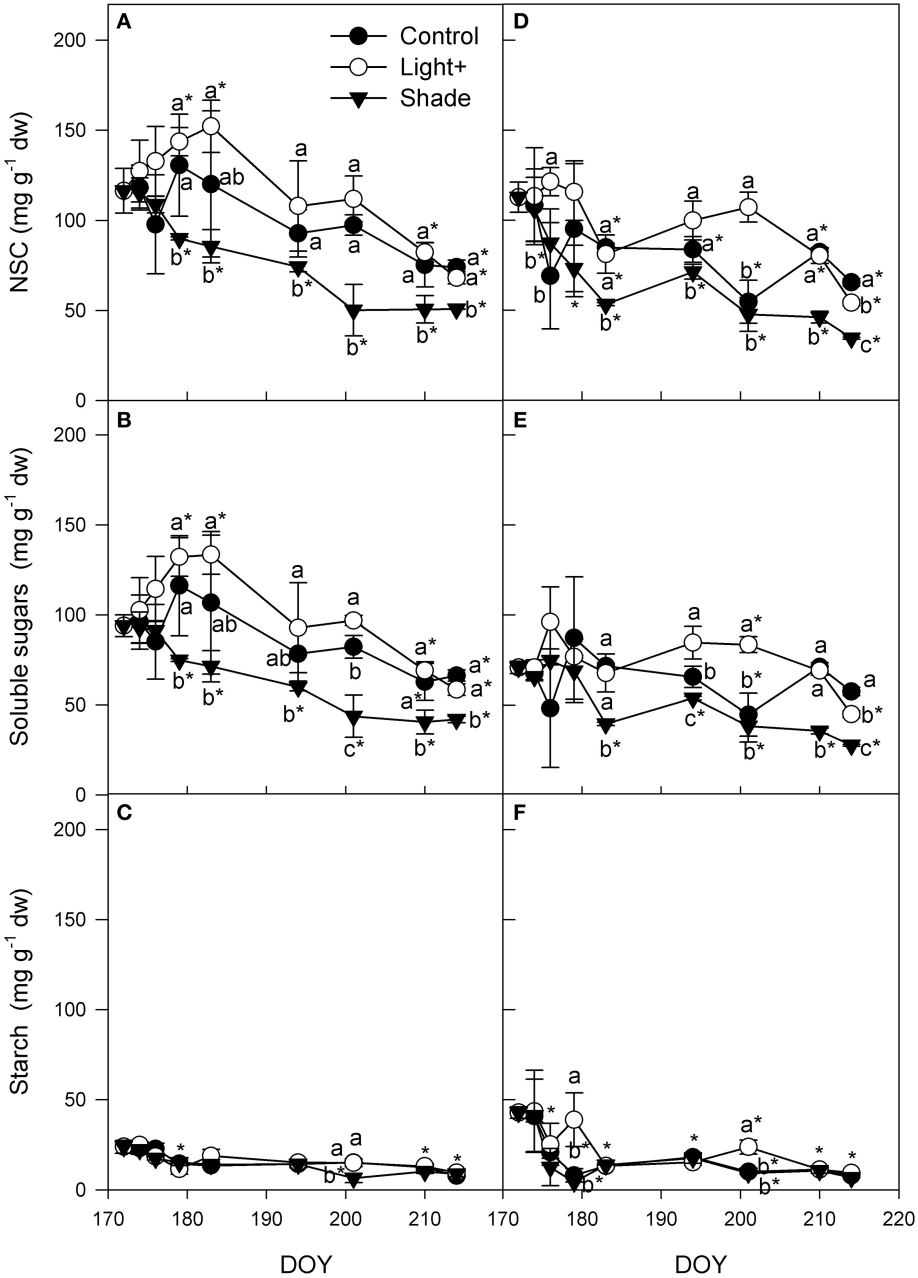
**Non structural carbohydrates (NSC), soluble sugars and starch in stems of cuttings collected from shoot apical part (A,B,C) and basal part (D,E,F)**. Each point is the mean of five stems ± SE. Points of the same sampling date with different letters are different per *P* = 0.05 (Tukey test). Points without letters were similar per *P* = 0.05. Point marked with asterisk are different from the value measured on the cutting collection day per *P* = 0.05 (Tukey test).

Stem NSC of cuttings collected from the apical part of the shoot increased in Light+ treatment until DOY 183, while in the same period it remained almost steady in Control treatment and decreased in Shaded treatment (Figure 7A). Thereafter, NSC content in stems decreased until the end of the experiment in apical cuttings of all treatments; Light+ and Control treatment kept a NSC content greater than Shaded treatment over the whole experiment. NSC pattern was mainly influenced by soluble sugars that had the same pattern described for NSC (Figure 7B). Starch varied little during the experiment even thought at the end it was significantly lower than the initial content in all three treatments (Figure 7C). Until DOY 179, stem NSC of basal cuttings was unchanged in Light+ treatment, while in the Control and Shaded treatment it decreased until DOY 183 (Figure 7D). Thereafter, NSC in Light+ and in Shaded treatment was almost unchanged until DOY 201 and then decreased again until the end of the experiment. In control treatment NSC decreased from DOY 183 until DOY 201. Generally speaking, Light+ treatment had greater NSC content than Control and Shaded cuttings, respectively, over the experiment. Soluble sugar content in stem of basal cutting was unchanged over the experiment in the Light+ and control treatments (Figure 7E) on the other hand soluble sugars decreased during the experiment in shaded treatment. Starch content decreased in all treatment of about 80% in the first 12 days since cutting collection (Figure 7F). Thereafter, starch content was almost unchanged until the end of the experiment (DOY 214).

**Figure 8:**
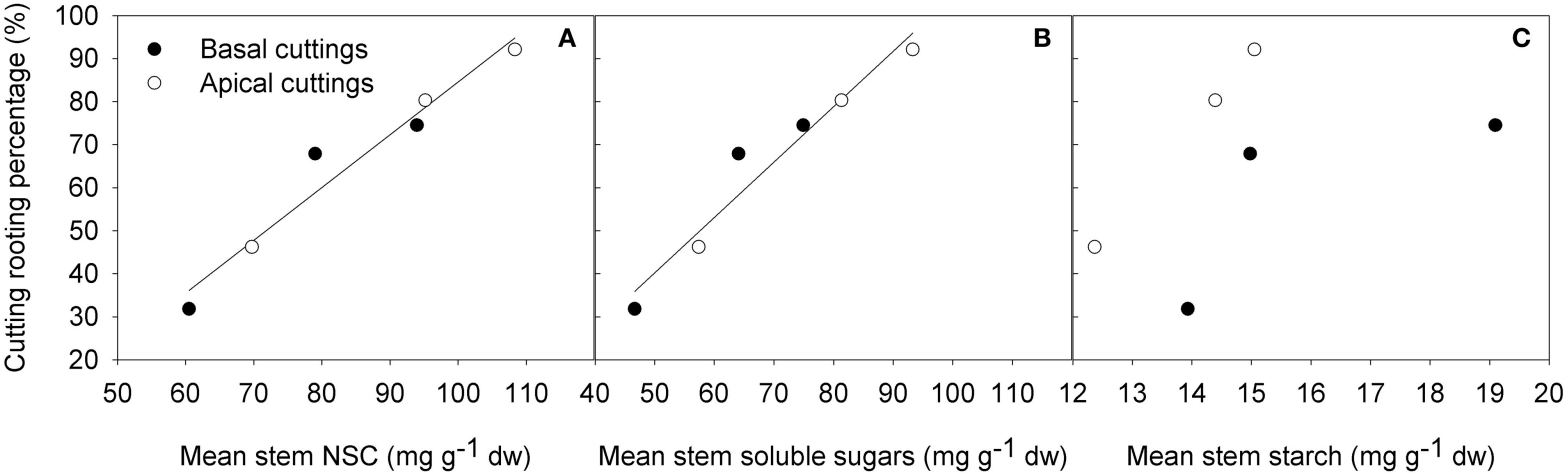
**Final cutting rooting percentages vs. mean NSC (A) (*y* = 1.22x-38.18 *R*^2^ = 0.95 *P* < 0.001), soluble sugars (B) (*y* = 1.29x-24.23 *R*^2^ = 0.94 *P* < 0.001), starch (C) content (*R*^2^ = 0.21 *P* = 0.36) in the basal part of the cutting stem during the whole period of rooting**.

After 22 days from cutting collection the large majority of cuttings were rooted or callused (Table 1). The Shaded treatment had the highest fraction of dead cuttings, whereas Light+ treatment showed the largest percentages of rooted and callused cuttings. Basal cuttings of the control treatment had the largest percentage of still cuttings followed by Shaded treatment cuttings. After 42 days from cutting collection, at the end of the experiment (DOY 214) light+ treatment had the highest percentage of rooted cuttings followed by Control and Shaded treatment, respectively (Table 2). Shaded treatment had the largest percentage of dead cuttings. Light+ had the highest percentage of well-rooted cuttings while in shaded treatment about half of the rooted cuttings had poor root formation and growth. In all treatments, cuttings from the apical part of the shoots showed better rooting than cuttings collected form the shoot apical part. Final rooting percentage was linearly correlated with the mean content of NSC and soluble sugars in the stem during the experiment (*R*^2^ = 0.95 *P* < 0.001, *R*^2^ = 0.94 *P* < 0.001, respectively) (Figures 8A,B). On the other hand final rooting percentage was not correlated with mean content of starch in the cutting stem during the experiment (*R*^2^ = 0.21 *P* = 0.36) (Figure 8C).

**Table 1:** **Relative frequencies of cuttings dead, still, callused and rooted at DOY 194 (22 days after cutting collection) in the three treatments**.

**Treatment**	**Cutting type**	**Dead**		**Still cuttings**		**Callused**		**Rooted**		**Callused** + **Rooted**	
Control	Apical	8.0%	e	27.1%	d	33.5%	c	31.4%	b	64.9%	c
	Basal	10.9%	c	41.8%	a	22.4%	e	24.8%	c	47.3%	d
Light +	Apical	4.1%	f	4.5%	e	43.5%	b	47.9%	a	91.4%	a
	Basal	22.3%	d	3.2%	e	47.9%	a	26.6%	c	74.5%	b
Shaded	Apical	25.4%	b	30.4%	c	26.4%	d	17.7%	d	44.1%	d
	Basal	39.7%	a	36.3%	b	15.0%	f	9.0%	e	24.0%	e

Relative frequencies with different letters in each column are different per P = 0.05 (χ^2^-test).

**Table 2:** **Relative frequencies of cuttings dead, poorly and well-rooted at the end of the experiment (DOY 214) after 42 days from cutting collection in the three treatments**.

**Treatment**	**Cutting type**	**Dead**		**Poorly rooted**		**Well-rooted**		**Total cuttings rooted**			
Control	Apical	19.7%	e	21.3%	b	59.0%	b	80.3%	b	74.5%	b
	Basal	32.1%	c	13.3%	d	54.5%	b	67.9%	c		
Light+	Apical	7.9%	f	9.6%	e	82.5%	a	92.1%	a	85.2%	a
	Basal	25.5%	d	26.1%	a	48.4%	c	74.5%	b		
Shaded	Apical	53.8%	b	16.7%	c	29.4%	d	46.2%	d	39.4%	c
	Basal	68.2%	a	16.9%	c	15.0%	e	31.8%	e		

Relative frequencies with different letters are different per P = 0.05 (χ^2^-test).

The highest post-rooting plant mortality occurred in plants of the Shaded treatment followed by those from Control and Light+ treatment, respectively (Table 3). In all three treatments, basal cuttings had larger mortality than apical cuttings. Plant mortality was larger in poorly rooted cuttings than in well-rooted cuttings in all treatments and independently by the portion of mother shoot from which cutting was collected.

**Table 3:** **Relative frequencies of plant mortality measured at March 31st 2015**.

**Treatment**	**Cutting type**	**Rooting quality**	**Plant mortality**					
Control	Apical	Well-rooted	27.7%	b	30.6%	b	33.9%	a
		Poorly rooted	37.5%	a				
	Basal	Well-rooted	35.3%	b	38.9%	a		
		Poorly rooted	50.0%	a				
Light+	Apical	Well-rooted	13.8%	b	17.0%	b	20.2%	c
		Poorly rooted	42.9%	a				
	Basal	Well-rooted	21.7%	b	27.1%	a		
		Poorly rooted	34.7%	a				
Shaded	Apical	Well-rooted	28.4%	b	30.6%	b	36.4%	b
		Poorly rooted	34.0%	a				
	Basal	Well-rooted	30.8%	b	46.5%	a		
		Poorly rooted	55.6%	a				

Relative frequencies with different letters are different per P = 0.05 (χ^2^-test).

## Discussion

Environmental conditions in the rooting bench where leafy cuttings are rooted are crucial for successful rooting In particular, leaf temperature and air VPD play a pivotal role in leaf survival and activity (Hartmann and Kester, 1983). In our experiment, the rooting bench was equipped with a mist apparatus that cooled leaves and decreased air VPD. All treatments were subjected to the same frequency of mist spray and as consequence of the different light regimes at which leafy cuttings were exposed, air temperature, RH, and air VPD varied depending on the treatment (Figure 1). In particular, in Light+, temperature increased and RH decreased in comparison with other two treatments resulting in higher values of air VPD that, in Light+, doubled as compared to the other two treatments. In theory, such environmental conditions are expected to be detrimental for cutting rooting, yet in our experiment we did not observe any increase of cutting mortality (Tables 1, 2) nor any decrease of leaf photosynthesis or stomatal conductance in Light+ cuttings in comparison with other two treatments.

During the experiment, water potential was consistently lower in Light+ cuttings than in Shaded cuttings. Cutting water potential was similar or even higher than that measured in the field prior cutting collection and stomatal conductance increased under greenhouse conditions. These data indicate that the environmental conditions imposed at cutting rooting period were adequate for leaf and cutting survival and that the used light regimes had a moderate effect on potential desiccation of hazelnut leafy cuttings.

Light saturation point measured in leaves prior cutting collection was around 500 μmol photons m^−2^ s^−1^ while maximum PAR during rooting was around 300 μmol photons m^−2^ s^−1^; at this and below this light intensity, photosynthetic efficiency was linearly correlated to PAR under field condition. In our experiment, leaf photosynthesis was linearly correlated with measured PAR. Cutting leaf photosynthesis was generally above or similar to that measured in the field prior cutting collection at similar PAR levels. Contrary to previous report on *Acer rubrum, Euforbia pulcherrima* and *Pinus contorta* (Smalley et al., 1991; Svenson et al., 1995; Brinker et al., 2004), our data suggest that in hazelnut cuttings leaf photosynthesis was not feedback down-regulated by carbohydrate accumulation in the leaf, and confirm the positive effect of environmental conditions (in particular low air VPD) on leaf photosynthesis.

Although cuttings collected from the apical part and basal part of the shoot had similar reaction to different light regimes during the experiment, apical cutting leaves had higher photosynthetic activity than leaves of basal cuttings. Furthermore, the increase of stomatal conductance over the rooting period was more marked in apical leaves than in basal leaves. These data suggest that apical leaves are more capable to supply carbohydrates to rooting cuttings than basal leaves. Apical leaves, developed at higher light intensity and slightly later than basal leaves, could be more photosyntetically efficient. These data are consistent with the larger stomatal conductance increase observed under low VPD in *Corylus maxima* full expanded leaves in comparison with younger leaves (Fordham et al., 2001). However, cutting mortality after rooting was consistently lower in apical cuttings than in basal cuttings (Table 3), indicating that reduced stomatal regulation was successfully counterbalanced by the acclimation technique used in the experiment.

The rooting process is a high demanding carbohydrates process and in our experiment the carbohydrate content in cuttings showed an overall decrease during the rooting process. The decrease was more evident in cutting stems, where intense metabolic processes related with root differentiation and growth occurs, rather than in cutting leaves (Figures 6, 7).

The increase of leaf photosynthetic activity, prompted by higher light intensity, caused an increase of NSC in leaves and, to a lesser extent, in stem base up to root formation (rooting occurred between DOY 183 and 194). After root formation, NSC in leaves and particularly in stems, constantly decreased confirming that root formation and elongation is a highly expensive process in the cutting carbon budget. These data also suggest that the first part of rooting process is less expensive for cuttings in terms of NSC. However, the poor photosynthetic activity measured in Shaded cuttings appeared to be not sufficient preserve NSC content in cuttings: in the first week from cutting collection, when callusing and rooting was still not detectable, NSC in Shaded cuttings decreased whereas in cuttings of the other treatments NSC content increased. Apical and basal cuttings followed approximately the same NSC pattern during rooting but with two major differences; although the spectacular increase (+42%) of NSC in Light+ leaves of basal cuttings was similar to that of apical cuttings subjected to the same light treatment, NSC in basal cutting stems did not increase. During the first 10 days after cutting collection, NSC remained steady even if starch decreased by 80% in the stem itself. The different pattern observed in cuttings collected from the basal and apical part of the shoot may suggest a different translocation rate of carbohydrates from leaves down to the basal part of the cutting stem: stems of basal cuttings did not reached the peak NSC level measured in stems of apical cuttings prior to rooting though NSC level in leaves was similar in basal and apical cuttings. Noteworthy, the percentage of cuttings callused or rooted after 22 days (at DOY 194) was almost equal to that of rooted in Light+ and Shaded treatments. In these two treatments the large variation of NSC between treatments appear to have played a pivotal role promoting or inhibiting adventitious root formation in the Light+ and in the Shaded treatment, respectively. The relatively lower NSC content, and in particular soluble sugar content, in stems of basal cuttings was in agreement with the consistent lower rooting recorded in stems of basal cuttings. Indeed, cutting rooting was linearly correlated with mean soluble sugar content in stems during rooting. These results suggest that once the hormonal promotion of adventitious rooting effectively occurred, cutting rooting in hazelnut is mainly driven by sugar availability in cutting stems. Thus, in hazelnut cutting leaf photosynthesis is an important factor during root initiation contrary to what generally reported in literature about photosynthesis and adventitious rooting (Davis, 1988).

Overall, the larger availability of NSC in stems had a positive effect on rooting quality and not just on rooting percentage; Light+ and Control cuttings had consistently more well-rooted cuttings than Shaded cuttings. Though, Light+ cuttings had consistently more NSC content than the other two treatments in the first week of the experiment, at the end of the experiment NSC content was similar across treatment; this can be explained as the effect of more root biomass grew in Light+ cuttings than in other treatments. Improved rooting quality in Light+ and Control treatments resulted in lower mortality in the post-rooting period. These results underline the importance of rooting biomass development for successful survival of rooted cutting that in hazelnut represent a critical point for economic viability of propagation by cuttings.

These results contribute to explain the contrasting effect of light intensities observed in many species in previous studies (Stoutemyer and Close, 1946; Waxman, 1965; Hansen, 1975; Loach, 1979; Loach and Gay, 1979; Grange and Loach, 1985; Aminah et al., 1997; Zaczek et al., 1997; Lopez and Runkle, 2008; Zobolo, 2010; Park et al., 2011; Currey et al., 2012). Light regimes below light saturation point can enhance photosynthetic activity of leafy cutting; however, excessive light regimes (above light saturation point for photosynthesis) can increase VPD and cause leaf desiccation without increasing leaf photosynthesis. Optimization of mist apparatus spray intervals can help to counterbalance undesired VPD increase caused by greater light regimes.

In conclusion, in hazelnut leafy cuttings, rooting appear to be closely related to carbohydrate, in particular soluble sugars, content in cutting stems during the rooting process. Cutting carbohydrate status is positively influenced by leaf photosynthesis during rooting. Moderate light regimes (below of light saturation point) of leafy cuttings can contribute to improve photosynthetic activity of leafy cuttings. Collection of cuttings from different part of the mother shoots influenced rooting percentage and this appear to be related to the lower capability of cuttings from shoot base of concentrating soluble sugars in the cutting stem during rooting. Finally, cutting rooting percentage and quality can be enhanced by optimization of light environment during rooting.

## Author contributions

ST and DF conceived and planned the study. ST and DF carried out the experiment, analyzed the data. ST wrote the first draft of the manuscript. AP, SP, and DF helped in the analysis of the data, revised, and edited the manuscript, and obtained funds to support the project.

### Conflict of interest statement

The authors declare that the research was conducted in the absence of any commercial or financial relationships that could be construed as a potential conflict of interest.
